# The Effect of Yoga Nidra Intervention on Blood Pressure and Heart Rate Variability Among Hypertensive Adults: A Single-arm Intervention Trial

**DOI:** 10.7759/cureus.77717

**Published:** 2025-01-20

**Authors:** Navdeep Ahuja, Monika Pathania, Latika Mohan, Sunita Mittal, Praag Bhardwaj, Minakshi Dhar

**Affiliations:** 1 Medicine, All India Institute of Medical Sciences, Rishikesh, Rishikesh, IND; 2 Physiology, All India Institute of Medical Sciences, Bilaspur, Bilaspur, IND; 3 Physiology, All India Institute of Medical Sciences, Rishikesh, Rishikesh, IND; 4 Faculty of Health and Wellness, Sri Sri University, Cuttack, IND

**Keywords:** autonomic nervous system, hypertension, interoception, lifestyle modifications, neurovisceral integration, parasympathetic system

## Abstract

Background

Hypertension [blood pressure (BP) >130/80 mmHg] contributes significantly to cardiovascular morbidity and mortality. Lifestyle modifications, including mindfulness-based practices like Yoga, meditation, and relaxation techniques, have emerged as promising adjuncts to pharmacotherapy. This study aimed to explore the acute effects of Yoga Nidra (YN) on BP in essential hypertension and the potential mechanisms of the effect of YN on BP, in the form of changes in heart rate variability (HRV) components.

Methods

A total of 32 hypertensive individuals (mean age: 43 ±0.54 years; 22 males, 10 females) were enrolled at the Lifestyle Disease Clinic. Patients were provided regular consultation and pharmacotherapy. BP and HRV were assessed before and after a 16-minute YN session. HRV parameters included time and frequency domain measures. Statistical analysis included linear regression to study the relationship of components of HRV with those of the changes in BP.

Results

Following YN intervention, there was a significant reduction in both systolic BP (SBP) (7 mmHg) and diastolic BP (DBP) (6 mmHg). HRV analysis revealed significant increases. Regression analysis showed changes in SBP having significant coefficients.

Conclusions

A single session of YN reduced the systolic and diastolic BP and increased HRV parameters. Regression analyses showed that the reduction in BP can be explained by an increase in HRV parameters. Thus, this study demonstrates the positive effect of YN as an intervention for essential hypertension and also the potential mechanisms behind it, which can be explained by the Neurovisceral Integration Model.

## Introduction

Burden of hypertension

The American Heart Association (AHA) defines hypertension as blood pressure (BP) of more than 130 mmHg for systolic BP (SBP) and 80 mmHg for diastolic BP (DBP) [1]. Around 31.1% of the total adult population is affected by hypertension worldwide [2]. The prevalence of essential hypertension is on the rise globally, doubling from 1990 to 2019 [3]. The number of adults with hypertension increased from 594 million in 1975 to 1.13 billion in 2015 [4]. This surge has largely been seen in low- and middle-income countries like India.

Cardiovascular diseases (CVDs) account for 17 million deaths a year, nearly 1/3 of the total [5]. Of these ailments, complications of hypertension account for 9.4 million deaths every year [3]. Proper control of hypertension could prevent more than 20-30% of the estimated 1.5 million deaths attributed to hypertension in India. It has been estimated that a 2 mm population-wide decrease in systolic blood pressure (SBP) in India would prevent 151,000 deaths due to coronary artery disease and 153,000 deaths due to stroke [6].

Pathophysiology of hypertension

Essential hypertension is caused by the interaction of numerous factors that alter the body's physiology. The most important factor is altered pressure natriuresis [7], in which the kidney’s capacity to excrete a given load of sodium ions is reduced at a given level of BP. As an adaptive mechanism, the sympathetic nervous system’s activity is increased and the BP rises, which allows the natriuresis of the sodium load and prevents excess sodium and fluid retention in the body. Thus, the balance of the sympathetic nervous system and parasympathetic nervous system is altered, resulting in hypertension [8].

Obesity also leads to sympathetic overdrive [9]. Dietary changes and weight loss are significant factors in reducing sympathetic drive. Hence, apart from antihypertensive medicines, lifestyle modifications have been recommended as an equal first-line approach for controlling hypertension [1]. In lifestyle modifications, relaxation techniques like yoga and meditation have been used to increase parasympathetic tone and reduce sympathetic activity, as well as reduce the renin-angiotensin system, which reduces BP. Nowadays, lifestyle modification is being emphasized to reduce stress levels and help lower BP using techniques like meditation, Tai Chi, Yoga, Qigong, etc [10].

Jennings et al. have published a review that investigates the causative role of stress and cognitive aspects in the development of hypertension [11]. They stated that the deficits in cognitive function, cerebral blood flow responsiveness, volumes of brain areas, and white matter integrity all relate to increased but prehypertensive levels of BP and that such relationships may be observed as early as childhood.

Role of lifestyle modifications

Physical activities like walking, cycling, Yoga, and transcendental meditation for stress management, dietary approaches to stop hypertension (the DASH diet), salt intake reduction, and alcohol reduction can all help in the treatment of hypertension as modifiable risk factors. Ebrahim et al. and many others have reviewed how lifestyle interventions reduce BP [12]. Vandana Yadav et al., in their meta-analysis on lifestyle modification and BP, reported the beneficial impact of these interventions in reducing both SBP and DBP. Physical activity resulted in a mean reduction of 3.46 mmHg in SBP and 1.28 mmHg in DBP. Weight reduction, a salt-restricted diet, the DASH diet, alcohol reduction, and Yoga caused a reduction in both SBP and DBP. Stress reduction in the form of transcendental meditation showed a mean difference of -4.9 mmHg in SBP and -3.0 mmHg in DBP [13].

Practices like alternate nostril Yoga breathing (ANYB) have been shown to reduce BP [14,15]. A single session of ANYB has been shown to significantly lower SBP and respiration rates by possibly increasing vagal activity during and after ANYB. This could have led to a drop in BP and changes in the heart rate variability (HRV) [15]. In a study by Huang et al., a single 90-minute session of Hatha Yoga decreased the low-/high-frequency (LF/HF) power ratio and scores on the perceived stress scale [16]. In another study, assessing the acute effects of a single Yoga session, the authors concluded that there is a healthy reduction in heart rate (HR), BP, and derived cardiovascular indices among geriatric subjects [17]. They attributed these changes to enhanced harmony of cardiac autonomic function as a result of coordinated breath-body work and mind-body relaxation following an integrated "Silver Yoga" program [17]. An objective measurement of cardiac autonomic nervous system activity can be done using HRV [18,19].

Role of Yoga Nidra and HRV

HRV is correlated with high BP in children [20]. In a study comparing subjects with prehypertension to those with normal BP, marked abnormalities in HRV parameters were observed. A meta-analysis of 17 studies deduced that reduced baroreflex sensitivity, believed to be secondary to increased arterial stiffness, is hypothesized to be implicated in the pathogenesis of essential hypertension [21]. The autonomic imbalance not only affects the BP but also has a negative impact on left ventricle (LV) dynamics [22]. In a landmark cohort study published in 2020 involving >29000 families, it was seen that heritability and genetics are related to both HRV and BP [23].

Yoga Nidra (YN) has been studied as a relaxing technique to relieve stress, and it was observed to significantly lower both systolic and diastolic BP in patients with hypertension who practiced the technique for 12 weeks [24]. In another study in older patients, a 15-day intervention entailing YN also lowered both SBP and DBP in patients with hypertension [25]. Thus, we opted for YN as an intervention to reduce the BP of patients with essential hypertension.

HRV is a significant marker of autonomic activity. HRV uses the beat-to-beat variability parameter and various types of analyses conducted on this parameter, such as linear and non-linear measures, to quantify the sympathetic and parasympathetic balance of the body. It has been demonstrated in Framingham Heart Study subjects that the LF and HF ratios of HRV are associated with hypertension in such a way that almost all HRV components were reduced in subjects having hypertension as compared to subjects having normal BP [18]. In addition, HRV can also predict the development of essential hypertension prospectively [18]. On cross-sectional analysis, HRV was significantly lower in hypertensive men and women. LF power, an HRV-derived parameter, was associated with incident hypertension in men. Yoo et al. have indicated that HRV indices are correlated with Framingham Heart Scores [19].

We aimed to look into the precise changes in autonomic functions brought about by a single session of YN in patients with essential hypertension. We hypothesized that if we can quantify the acute changes in cardiovascular physiology while performing the intervention of YN, it could be used to quantify the changes induced by different sessions and to follow the long-term changes in those parameters. Also, it could be used as a prognostic indicator of future CVD risk and a reduction in complications of essential hypertension, such as coronary artery disease, stroke, and renovascular complications. Thus, we chose HRV to measure autonomic activity during and after an intervention involving a single session of YN in hypertensive patients. Our primary objective was to measure the effects of YN intervention on BP; the secondary objectives were to measure the effects of YN intervention on HRV and to assess whether changes in HRV can be explained as the potential mechanisms for this change in BP brought about by the YN intervention.

## Materials and methods

Recruitment of patients

The study was conducted as a single-arm pilot study. Ethical clearance was obtained from the Institutional Ethics Committee (number AIIMS/IEC/22/494), and the trial was registered in the Clinical Trials Registry of India (CTRI/2023/01/048719). Participants were recruited from the Lifestyle Disease Clinic OPD, Department of Internal Medicine, AIIMS Rishikesh with a convenience sampling method. Written informed consent was obtained from all participants before the study.

**Figure 1:**
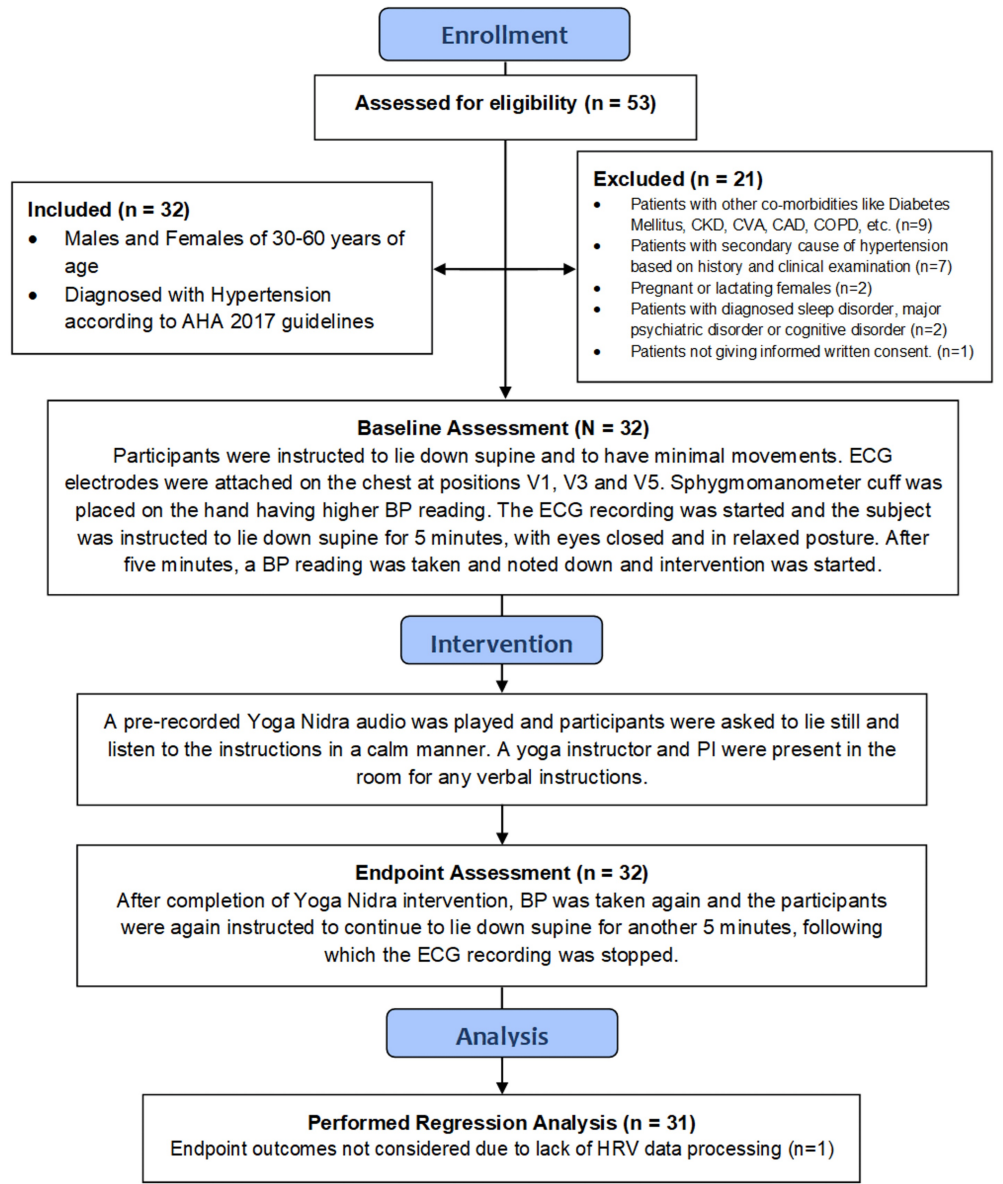
Flow diagram showing a brief overview of the study methodology AHA: American Heart Association; BP: blood pressure; CAD: coronary artery disease; CKD: chronic kidney disease; COPD: chronic obstructive pulmonary disease; CVA: cerebrovascular accident; ECG: electrocardiogram; HRV: heart rate variability; PI: principal investigator

The study population comprised 30 to 60-year-old patients diagnosed with hypertension according to AHA 2017 guidelines. Participants with any other comorbidities such as diabetes mellitus, chronic kidney disease, coronary artery disease, and chronic obstructive pulmonary disease were excluded from the study. Also, participants having a secondary cause of hypertension, pregnant/lactating females, participants diagnosed with sleep disorders or major psychiatric disorders, and participants not willing to give informed consent were excluded. The sample size consisted of a total of 32 participants. The patients were given standard care and pharmacotherapy in addition to the YN intervention. Figure 1 presents an overview of the study methodology followed for each subject.

Procedure

Participants were instructed to avoid taking caffeine, nicotine, or alcohol 24 hours before the procedure, to avoid food intake for two hours prior, and to empty the bladder. A resting BP was taken in both the arms and the arm having a higher BP was used subsequently for the measurements. During the procedure, participants were instructed to lie down supine in the dimly lit Yoga demonstration room on a Yoga mat. Participants were instructed to have minimal movements during the procedure. To document the HRV, a Holter device (Contec TLC9803 Holter ECG system, Qinhuangdao, China) was used to record the ECG during the intervention period. The ECG electrodes were attached to the chest at positions V1, V3, and V5. A sphygmomanometer cuff was placed on the arm with a higher BP reading. The ECG recording was started, and the subject was instructed to lie down supine for five minutes with eyes closed and in a relaxed posture. After five minutes, a BP reading was taken and noted down.

**Figure 2:**
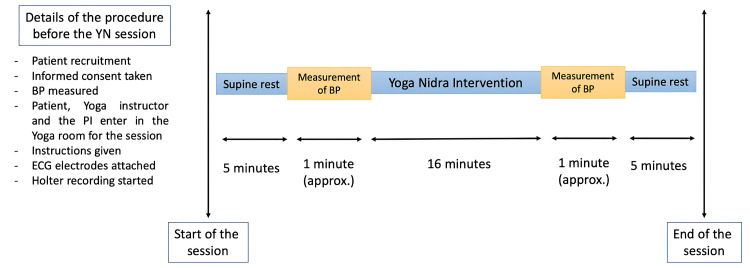
Details of the YN intervention session conducted for each patient BP: blood pressure; ECG: electrocardiogram; PI: principal investigator; YN: Yoga Nidra

For the YN intervention session, participants were instructed beforehand, in detail, about the position to be maintained while performing YN, and how they will be guided in paying attention to different parts of the body. To prevent intra-observer variability, a pre-recorded audio file of guided YN instructions was played, in the voice of the widely acknowledged Yoga Master, Sri Sri Ravi Shankar after obtaining due permission from the Sri Sri Institute for Advanced Research (SSIAR). The audio consisted of a series of verbal instructions including conscious relaxation and sequential rotation of consciousness through different parts of the body [26]. The intervention lasted for approximately 16 minutes. At each step, time points were noted with the help of a stopwatch. Figure 2 depicts the details of the YN protocol.

A well-qualified Yoga teacher and evaluator along with the PI were present in the room to supervise the participant and provide any verbal assistance if required. In the case of female participants, a female nurse or attendant was present in the room along with the PI to ensure their comfort and safety. After the completion of the YN intervention, BP was taken post-intervention again, and the participants were again instructed to continue to lie down supine for another five minutes for recording of ECG data for obtaining the HRV. At the end of five minutes post-intervention, the electrodes were removed and the subject was asked to sit up slowly. Participants continued to take standard treatment along with a regular YN practice after the session.

Data analysis

ECG Data

The ECG data was manually screened for any artifacts in the software provided along with the Holter equipment. The RR intervals were generated from the lead having artifact-free RR intervals in a file separately for all subjects. The RR Interval file was imported in MATLAB version R2023a [27]. Also, the time interval file was created to specify the time points for which HRV was done in windows of 300 seconds. For HRV analysis, the Physionet toolbox was used [28,29]. The RR Interval files were used to generate both time-based and frequency-based parameters. HRV parameters were calculated for sequential five-minute intervals taken during the first 15 minutes of the 16-minute period of the intervention. Thus, the HRV was calculated for these periods: the pre-intervention rest period; three intervals of five minutes each during the intervention during the first 15 minutes of the recording; and the post-intervention rest period. BP was taken with a calibrated sphygmomanometer using the auscultatory method.

Linear Regression

Linear regression was performed with the dependent variables as the change in systolic and diastolic BP. Independent variables assessed were as follows: age, gender, pre-intervention SBP, pre-intervention DBP, and each of the different parameters of HRV [HF, LF, standard deviation of all normal sinus RR intervals (SDNN), root mean square of successive differences (RMSSD), the percentage of successive normal sinus RR intervals >50 ms (pNN50), LF/HF ratio, NN mean, and total power] at different time points (pre-intervention, during the intervention, and post-intervention), and change after the intervention, thereby generating a total of 36 variables.

*Statistical Analysis* 

The data was tabulated in an Excel sheet and analyzed with the help of MATLAB software. Taking the confidence level as 95%, the statistical significance was set at p<0.05.

BP: At first, the distribution was checked for normality with the K-S test, and then a paired t-test was applied.

HRV parameters: At first, the distribution was checked for normality with the K-S test, and then a related sample Wilcoxon signed-rank test was applied as all the parameters had a non-normal distribution.

Regression analysis was conducted in MATLAB with the function stepwiselm in MATLAB’s Statistical and Machine Learning Toolbox.

## Results

Patient characteristics

A total of 32 participants were recruited for this study. The HRV data for one subject could not be processed. Hence, BP data from 32 subjects and HRV data from 31 subjects were used. The mean age of the participants was 43 ±0.54 years. The mean BP of participants at baseline was 143 ±17 (systolic) and 94 ±12 (diastolic BP) mmHg. There were 22 males and 10 females in the cohort.

BP and HRV

**Figure 3:**
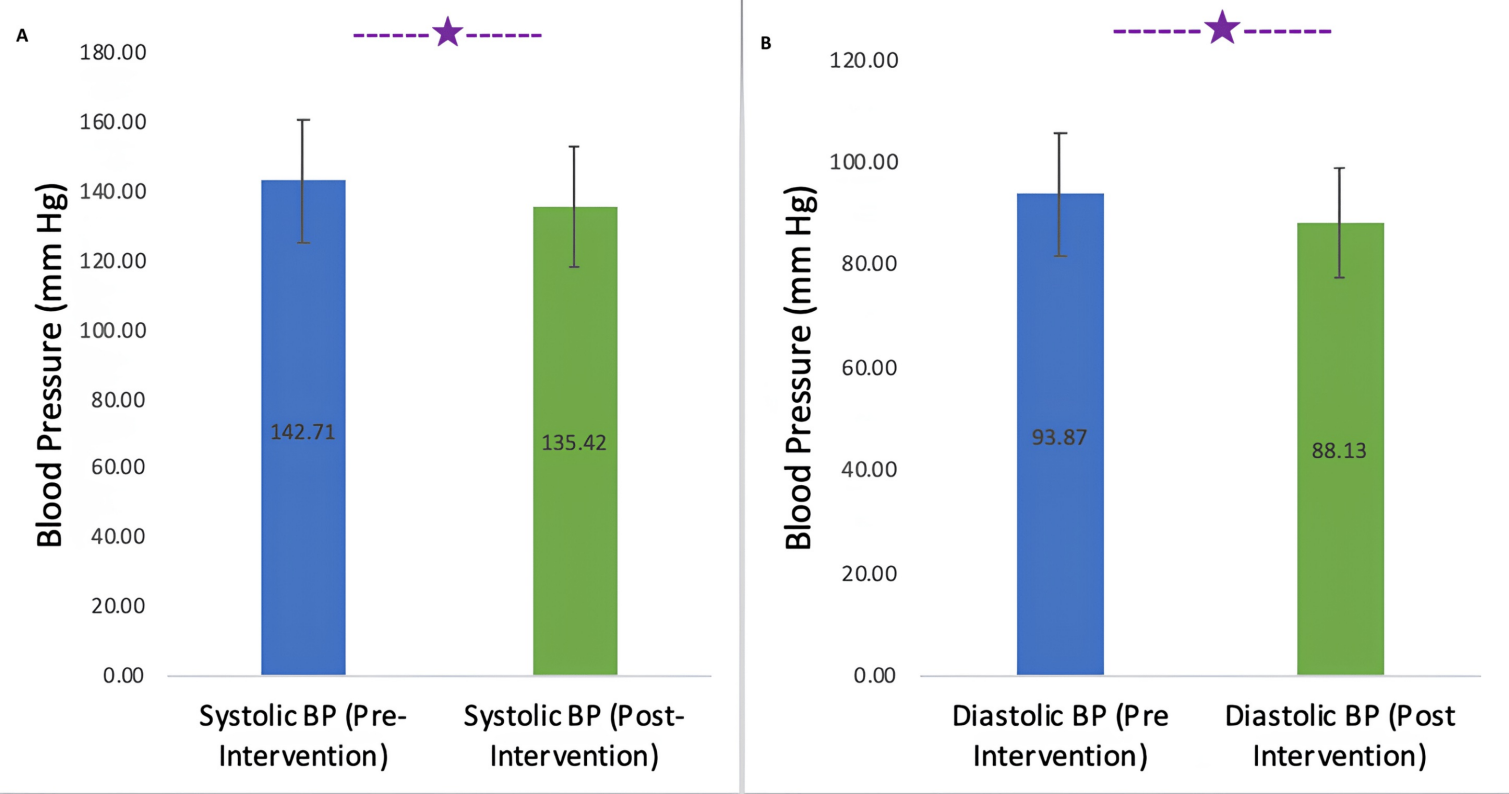
Bar graphs showing pre-intervention and post-intervention BP *P<0.05 A: Pre-intervention and post-intervention systolic BP. B: Pre-intervention and post-intervention diastolic BP BP: blood pressure

After the intervention, there was a statistically significant change in both systolic and diastolic BP (p<0.001 for both SBP and DBP). The mean difference in SBP was 7.12 ±6.46 mmHg while that in DBP was 6 ±5.27 mmHg. The resultant effect size for paired values was (1.1). Figure 3 depicts the SBP and DBP values before and after the YN intervention.

**Figure 4:**
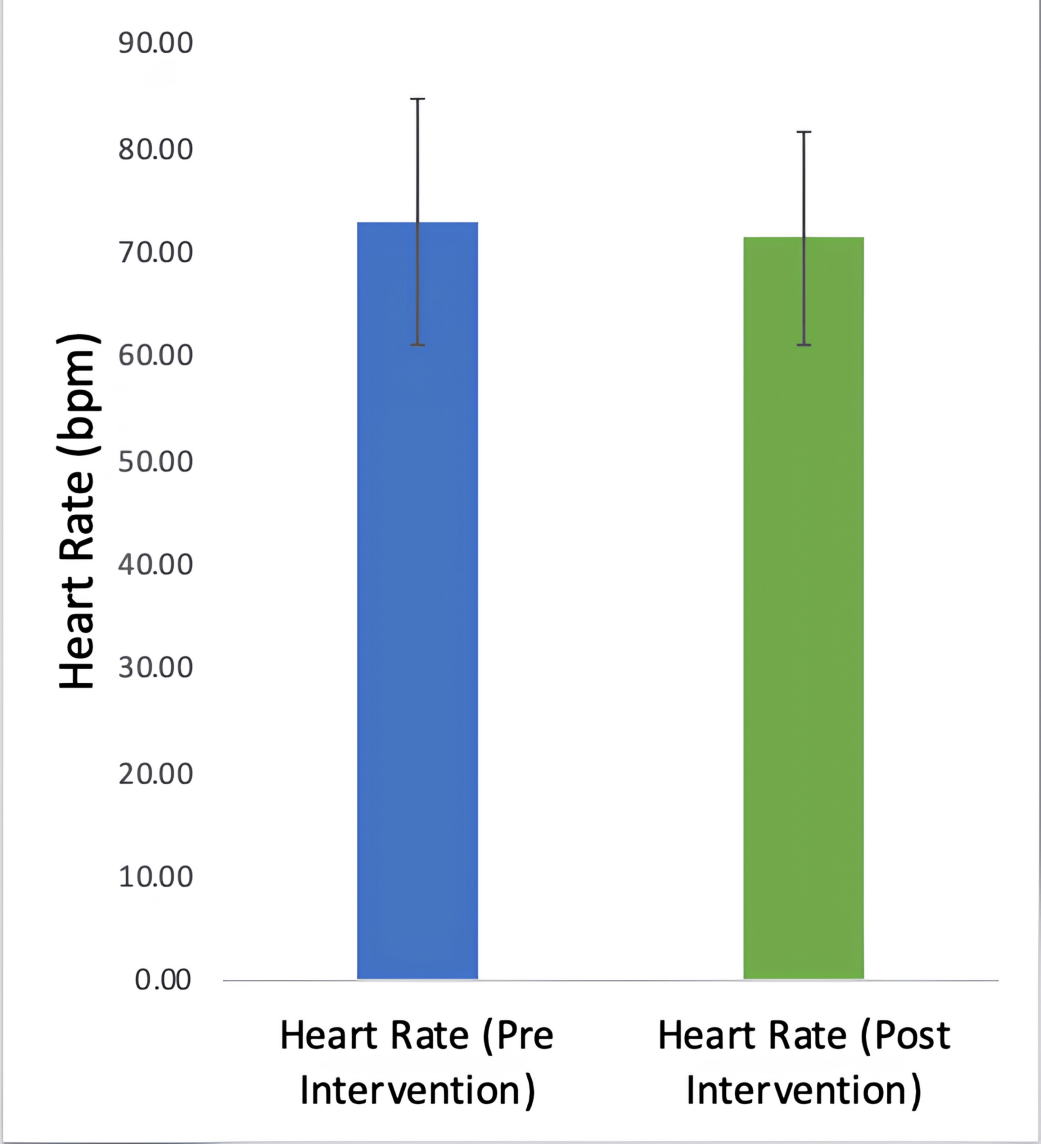
Bar graph showing pre-intervention and post-intervention heart rate

The heart rate before the intervention was 72.83 ±11.89 bpm, which was not significantly different from the heart rate after the intervention: 71.34 ±10.2 bpm. Figure 4 depicts the heart rate before and after the YN intervention.

The HRV data were further classified into time and frequency parameters. The time HRV parameters were SDNN, RMSSD, and pNN50, and the frequency parameters were HF, LF, and LF/HF ratio. A signed rank test between pre and post-intervention variables demonstrated the following p values: LF: 0.0037; HF: 0.004; LFHF: 0.5435; total power: 0.0187; SDNN: 0.0077; RMSSD: 0.012; pNN50: 0.0938.

**Figure 5:**
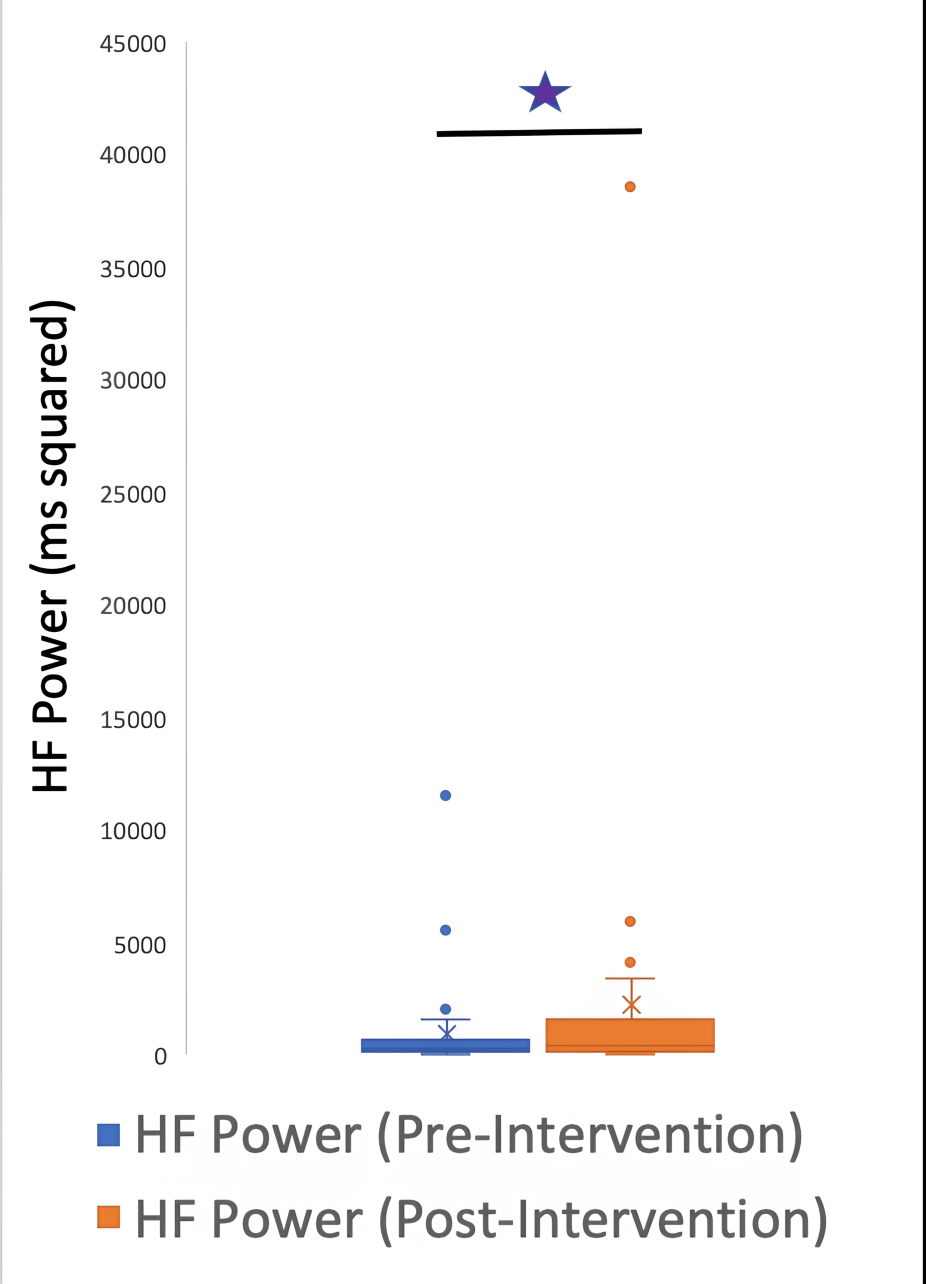
Box plot showing the HF power before and after the intervention *P=0.004 HF: high-frequency

**Figure 6:**
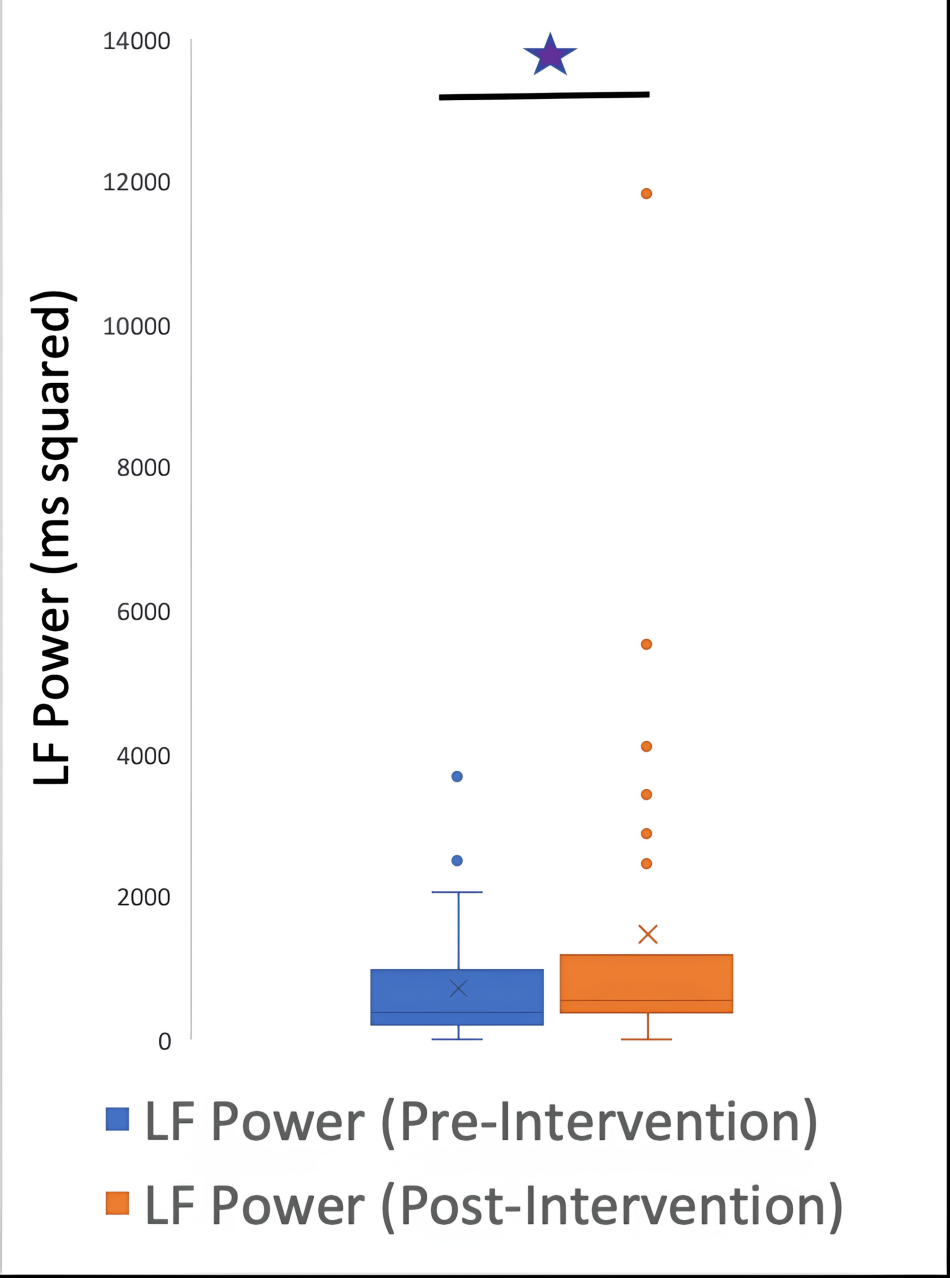
Box plot showing the LF power before and after the intervention *P=0.0037 LF: low-frequency

**Figure 7:**
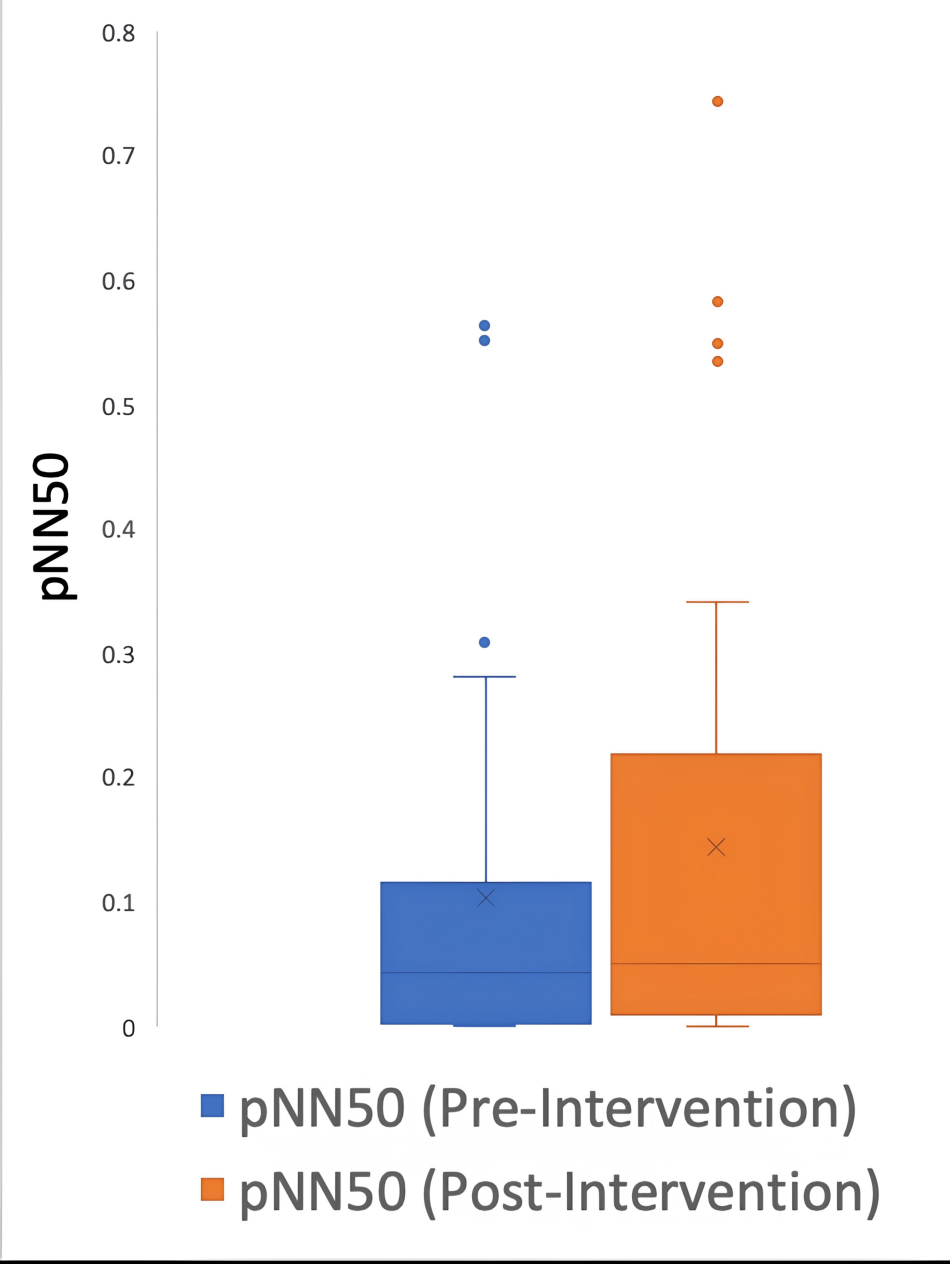
Box plot showing the pNN50 power before and after the intervention P=0.09 pNN50: proportion of NN50 divided by the total number of NN (R-R) intervals. NN or R-R intervals represent the time between two successive heartbeats

**Figure 8:**
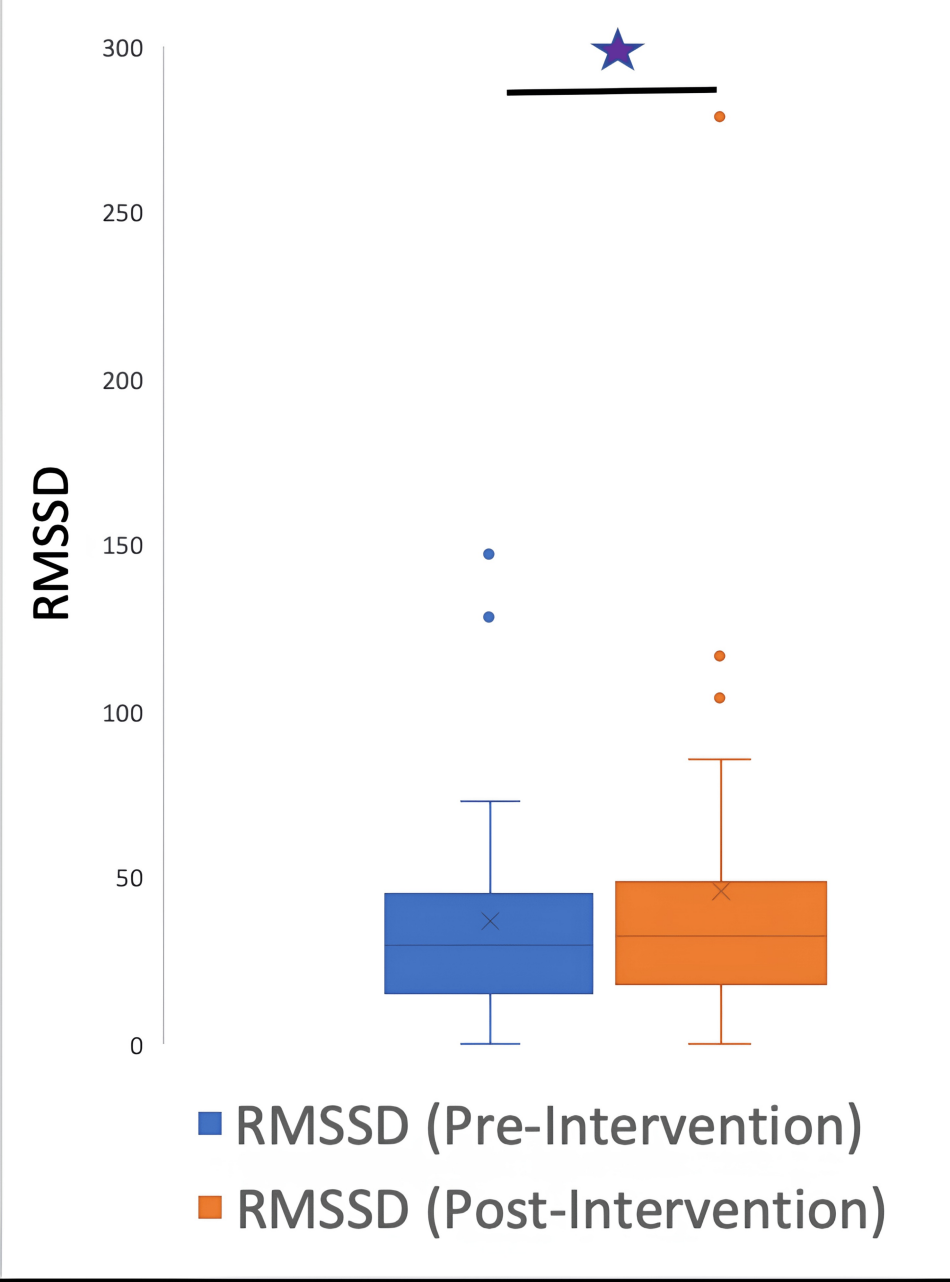
Box plot showing the RMSSD before and after the intervention *P=0.012 RMSSD: root mean square of successive differences

**Figure 9:**
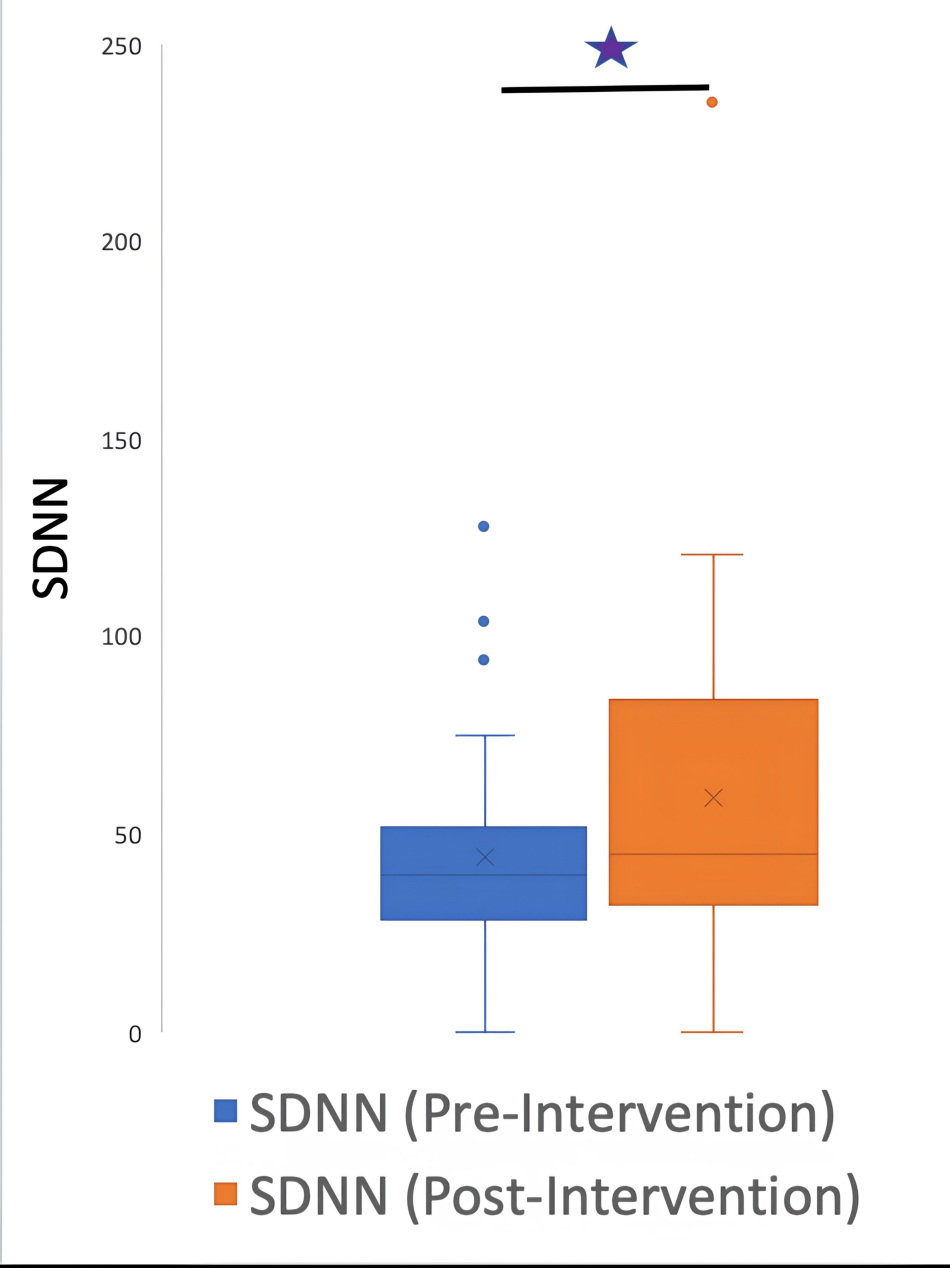
Box plot showing the SDNN before and after the intervention *P=0.0077 SDNN: standard deviation of the NN (R-R) intervals. NN or R-R intervals represent the time between two successive heartbeats

**Figure 10:**
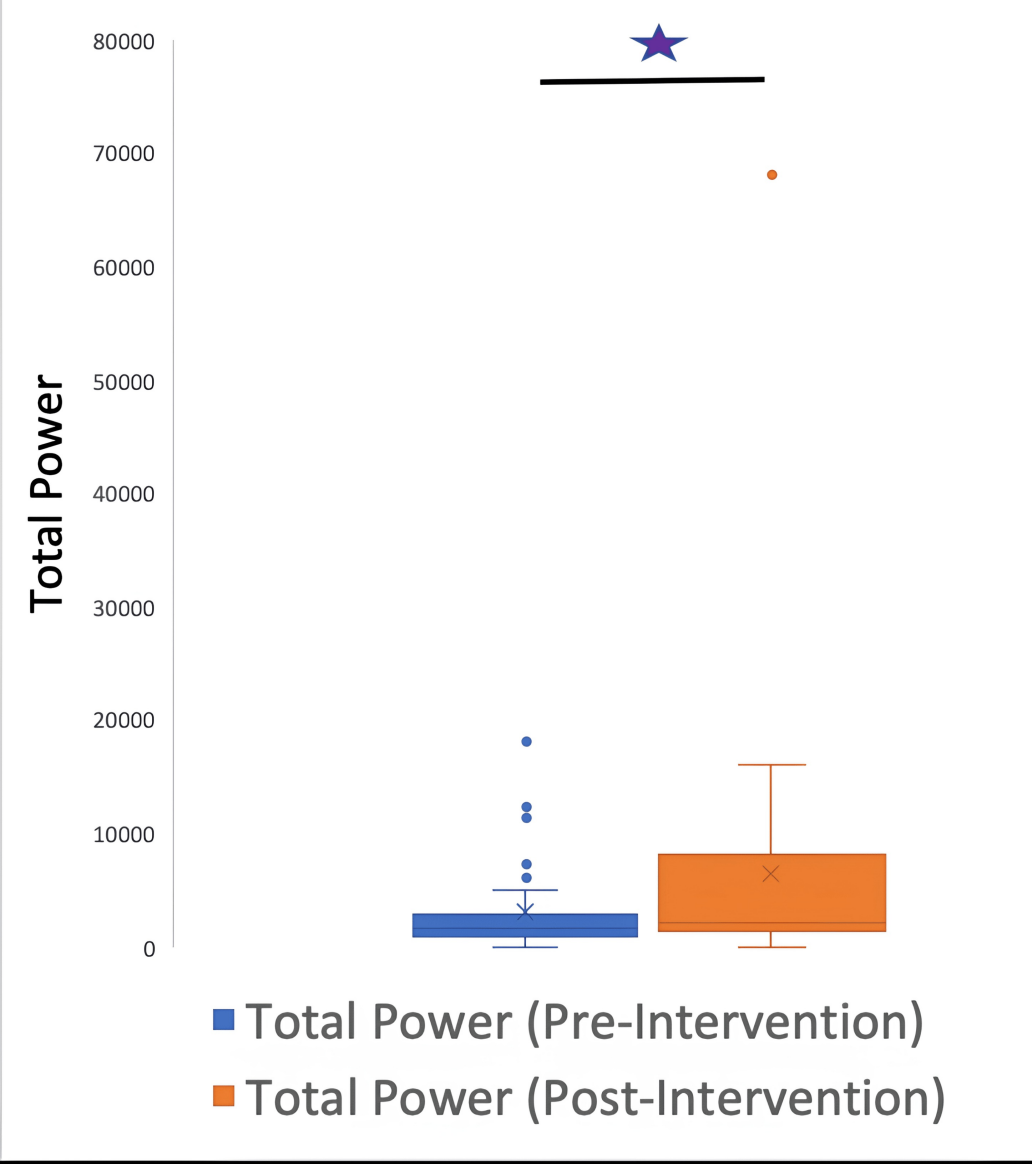
Box plot showing the total power before and after the intervention P=0.018

The parameters LF, HF, total power, SDNN, and RMSSD underwent a significant change after the intervention; however, the LF/HF ratio and pNN50 did not have a significant change. Figures 5-10 show the box plots for the different parameters of HRV before and after the intervention.

Linear regression

**Figure 11:**
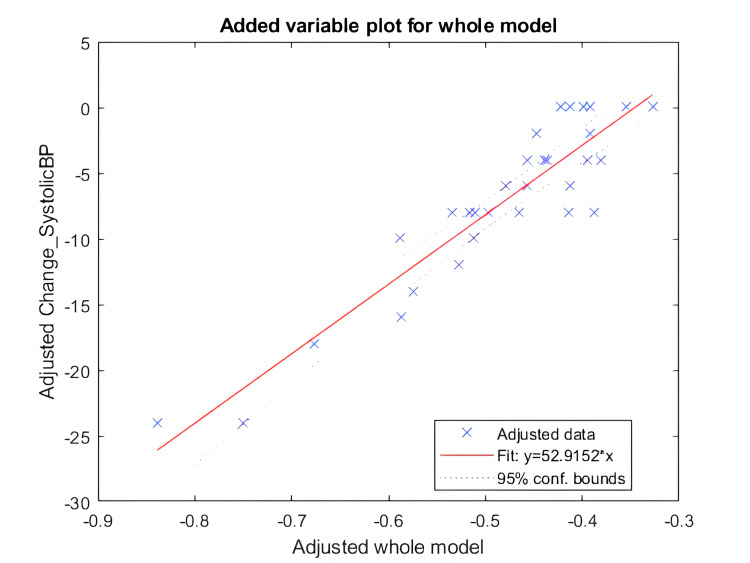
Scatter plot for the linear regression model fit for change in systolic BP The X-axis shows the coefficients of the whole model, and the Y-axis shows the change in systolic BP in mmHg BP: blood pressure

The change in SBP has significant regression coefficients with respect to independent variables, namely, pre-intervention SBP, pre-intervention DBP, change in pNN50, change in LF, and change in RMSSD. The coefficients for pre-intervention DBP and change in pNN50 were negative (-0.4 and -52, respectively), whereas the coefficients for pre-intervention SBP, change in LF, and RMSSD were positive. Figure 11 shows the results of linear regression in the form of a scatter plot.

**Figure 12:**
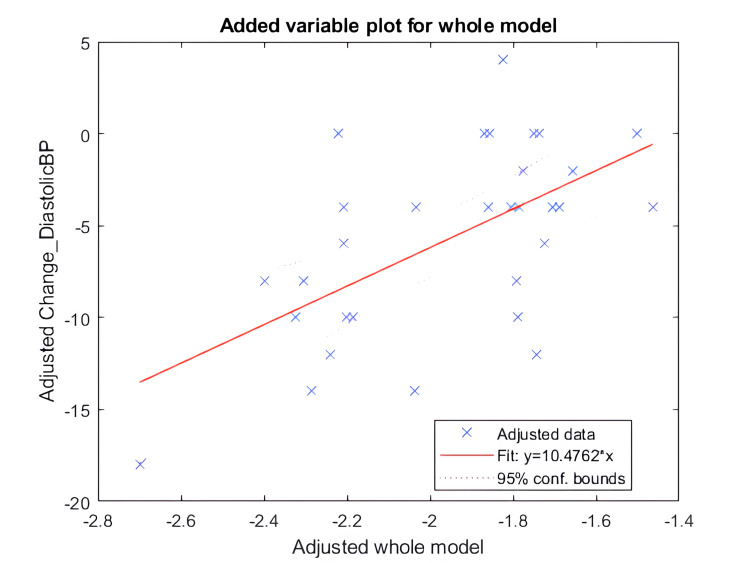
Scatter plot for the linear regression model fit for change in diastolic BP The X-axis shows the coefficients of the whole model, and the Y-axis shows the change in diastolic BP in mmHg BP: blood pressure

The change in DBP has significant regression coefficients with independent variables, namely, pre-intervention DBP and post-intervention pNN50. The coefficients for both variables were negative (-0.2 and -10, respectively). Figure 12 shows the results of linear regression in the form of a scatter plot.

## Discussion

Summary

This study's results reveal a statistically significant decrease in BP following a single session of a 16-minute YN intervention. Our second objective was to study the putative mechanisms responsible for the change in BP. The study found an increase in HRV parameters after the intervention. This result could be interpreted as an elevation in parasympathetic activity induced by YN, which, in turn, plays a significant role in the observed reduction in BP.

To further understand and evaluate the mechanism, we performed linear regression analysis to predict changes in both SBP and DBP based on various independent variables. It's important to note that BP change is calculated as the difference between post-intervention BP and pre-intervention BP, with a negative value indicating a reduction in BP. A negative coefficient for an independent variable signifies its propensity to favor a reduction in BP post-intervention.

Regarding SBP, we observed that the coefficients for pre-intervention DBP and the change in pNN50 were negative (-0.4 and -52, respectively), while the coefficients for pre-intervention SBP, change in LF, and change in RMSSD were positive. This suggests that higher pre-intervention DBP and a more substantial change in pNN50 tend to result in a reduction in systolic BP. Conversely, higher pre-intervention SBP does not exhibit a similar reduction effect. It is worth noting that the coefficient for the change in DBP is -0.4, whereas the coefficient for the change in SBP is 0.14, with the latter having a smaller magnitude. Therefore, overall, a higher DBP appears to have a more pronounced impact on reducing SBP compared to the opposing influence of a higher SBP.

Based on the outcomes of the linear regression analysis, a putative inference is that pNN50 plays a pivotal role in BP reduction. pNN50 is known to increase with heightened parasympathetic activity and is reduced in individuals with hypertension [5]. Although the change in pNN50 was not significant in our study, it showed a trend toward an increasing direction in the post-intervention state.

Mechanisms of the effect of YN on BP and HRV

YN enhances parasympathetic activity through complex cognitive mechanisms. One model is the Neurovisceral Integration Model described by Thayer et al. [30], which outlines the role of higher-level cortical regions in integrating emotional responses with autonomic functions. HRV serves as an indirect marker of attention and behavioral regulation within this framework.

Smith et al. have proposed an eight-level hierarchy that acts as a bridge between higher cortical regions and the centers directly influencing cardiac autonomic activity [31]. The initial three levels encompass the brainstem and regions like the nucleus tractus solitarius (NTS), which directly modulate heart rate and, consequently, affect HRV through both parasympathetic and sympathetic pathways. However, the higher levels of this hierarchy include structures that can exert direct control over HRV or indirectly influence it through top-down regulation of other brain regions.

These higher-level areas comprise the hypothalamus, periaqueductal gray (PAG), amygdala, basal forebrain, insulin, cingulate cortex, medial prefrontal cortex, and frontoparietal cortex. The hypothalamus coordinates autonomic, endocrine, and behavioral responses to emotional stimuli. The amygdala detects threatening stimuli via its connections to the cortex. Other areas, such as the insula and cingulate cortex, contribute to the interpretation of perceptual input and the representation of somatic and visceral states. The medial prefrontal cortex assesses the emotional significance of a situation, while the dorsolateral prefrontal cortex contextualizes affective information within an individual's current goals. Collectively, this hierarchical network of brain regions, either directly or indirectly, plays a role in HRV. These are the possible mechanisms by which interoception during YN affects the autonomic nervous system and consequently HRV, which would require further investigation.

Effects of Yoga practices on BP and HRV

Yoga practices have demonstrated the capacity to enhance HRV. This is supported by a meta-analysis that has shown the positive influence of Yoga on HRV [32]. Furthermore, exercise has also been linked to favorable HRV outcomes [33]. Sharpe et al. showed that the RMSSD increased with deep breathing, one of the techniques in Pranayama [34]. Given that YN also incorporates elements of deep breathing, it presents a potential avenue for increasing HRV. Anasuya et al.'s work has demonstrated elevated HRV among Yoga practitioners [35]. Furthermore, Benvenutti et al.'s findings indicate a reduction in stress reactivity following Yoga practice [36]. These collective insights underscore the favorable impact of Yoga, including its components like deep breathing and relaxation, on HRV.

According to the Framingham Study, HRV is a useful prognostic marker. Angiotensin II-driven elevated renin activity, which in turn causes increased sympathetic outflow centrally, has been associated with decreased HRV [37]. Telles et al. have undertaken studies that demonstrate changes in HRV and BP by employing Anulom Vilom Pranayama (ANYB), a Yogic technique that can influence central autonomic functions [14,15]. These investigations have significantly advanced our understanding of how such practices can impact physiological parameters. In a similar vein, Goit et al. have contributed valuable insights by demonstrating reduced HRV in individuals with hypertension [38]. Their findings closely parallel our own, further corroborating the link between HRV and hypertension and underscoring the importance of our research.

Effects of YN on BP

Previous studies have shown that YN causes a reduction in BP in hypertensive patients [39-41]. The YN intervention in these studies lasted for a few weeks (2-24 weeks). Thus, it has been shown that YN is an effective intervention for hypertension when practiced at regular intervals over a relatively longer duration. However, in the current study, we have shown that a single session of YN itself can cause a reduction in BP, which is likely mediated through a central increase of parasympathetic drive which in turn increases the HRV measures and eventually leads to a reduction in BP.

Effects of a single session of Yoga on BP and HRV

Our choice of a single session of intervention was deliberate, allowing us to observe its immediate effects and discern its mechanisms. A single session of Silver Yoga has shown promise by effectively reducing BP [17]. Thanalakshmi et al. conducted HRV assessments during the administration of Sheetali Pranayama as an intervention [42]. However, it remains to be determined to what extent the effects of such single sessions are useful in the long term. Previous research has indicated that after several weeks of consistent yoga practice, a notable reduction in BP is observed. Based on these findings, we advocate for a comprehensive assessment of YN, to incorporate evidence-based approaches into hypertension management guidelines.

Future directions

Our research paper presents evidence of the acute impact of a single YN session on systolic and diastolic BP, both of which exhibited statistically significant reductions. Furthermore, our study goes beyond the mere observation of BP changes and delves into the potential mechanisms underlying these alterations. Specifically, we investigated the influence of YN on HRV components, which is a major strength of this study and a novel field of investigation. The observed increase in HRV parameters suggests a shift toward increased parasympathetic activity and a reduction in sympathetic activity. This shift is indicative of YN's potential to modulate the autonomic nervous system, a plausible mechanism contributing to the observed BP reduction. To further understand the mechanism, we conducted linear regression analyses, which revealed that some HRV components can explain the variability in BP reduction. This statistical approach adds weight to the hypothesis that changes in HRV mediate the acute BP-lowering effects of YN. However, more studies are required to conclusively understand the mechanisms behind such an effect.

However, it is important to acknowledge that our study primarily addressed acute effects, which is a limitation of the study, and we recognize the necessity for further investigations. Future research endeavors should aim to ascertain whether these acute effects persist over extended periods, possibly due to the neuroplasticity of higher cognitive areas. Such plasticity could influence the autonomic nervous system, HRV, and BP regulation.

Our findings show that HRV is a valuable parameter for quantifying cardiovascular health by estimating the activity of the sympathetic and parasympathetic branches of the autonomic nervous system. The assessment of HRV in response to interventions such as YN represents a crucial step in understanding the potential benefits of mind-body practices in the context of health management. However, to move beyond anecdotal claims and truly utilize the impact of these interventions, we must subject them to rigorous quantification techniques.

Limitations

This study has a few limitations, including a lack of a control arm in the trial. Also, similar recordings of the BP and HRV could be measured over a few weeks to demonstrate a lasting effect on BP, which was not performed in this study. However, such studies could be conducted in the future considering the significant results of the current study. Another limitation of the current study is its relatively smaller sample size.

## Conclusions

YN is an effective intervention for essential hypertension and it acts by increasing the parasympathetic activity, as measured by HRV, and leads to a reduction in BP. It has been shown to reduce BP when performed for a few weeks. However, our findings show that even a single session of YN leads to a reduction in BP. Thus, YN is a cost-effective intervention, freely available to all patients, which can be specifically helpful in resource-limited settings, a significant point to be considered in the context of pharmacoeconomics of the management of essential hypertension at the community level. Also, it can have larger positive implications in terms of reducing the significant burden of CVDs on the population, if utilized in the right manner.
